# Peer review of "Volatilome Analyses and In Vitro Antimicrobial Activity of the Essential Oils from Five South African Helichrysum Species"

_molecules, 2020, doi:10.3390/molecules25143196_

Round 1

Reviewer 1 Report

The article submitted by Basma Najar et al, described the analysis of the volatile molecules of five new South African species of Helichrysum and their antimicrobial and antimitotic effect.

The article is suitable for Molecules. The instrument used are adequate and the statistical tests are appropriate.

In my opinion, the authors described an interesting GC-MS approach to unravel new essential oils.

However, there are some flaws surrounding this piece of work, which require changes in the presentation of the results.

  • Figure legends are swapped for figure 1 and 2 and figure 3 and 4.
  • I would suggest to check English spelling and removing Italian word ‘Classe’, ‘totale identificato’ in table 2.
  • The numbering of the table is according to their appearance in the text. Table 1 is at the end of the article. Please change.
  • It is unclear the variation in the table 2, please provide full legend e.g. ‘H. cooperi’ (percentage%) and define percentage of identification.
  • Please provide more information in the figure legends.
  • I would suggest to separate in new table the chemical class from the identified compounds.
  • Please report references for the Table 4 and 5 for previous studies.
  • Table 1, which is at the end, please put as supplementary information.

In my opinion, the authors should not underestimate their results and add few more information.

  • The manuscript will benefit if the authors will add in the table, molecular weight and elemental composition of the identified metabolites.
  • It would be beneficial a Venn diagram where readers can appreciate the difference of the composition of the five species.
  • Please provide in the supplement the representative total ion chromatograms for the 5 species.
  • In my opinion the manuscript will benefit from few chemical structures of the mostly changes metabolites.
  • Please provide a pie chart or bar chart to illustrate the difference in compositions.

Author Response

Reviewer 1

Open Review

English language and style

( ) Extensive editing of English language and style required 
( ) Moderate English changes required 
(x) English language and style are fine/minor spell check required 
( ) I don't feel qualified to judge about the English language and style 

Yes

Can be improved

Must be improved

Not applicable

Does the introduction provide sufficient background and include all relevant references?

(x)

( )

( )

( )

Is the research design appropriate?

( )

(x)

( )

( )

Are the methods adequately described?

(x)

( )

( )

( )

Are the results clearly presented?

( )

(x)

( )

( )

Are the conclusions supported by the results?

(x)

( )

( )

( )

Comments and Suggestions for Authors

The article submitted by Basma Najar et al, described the analysis of the volatile molecules of five new South African species of Helichrysum and their antimicrobial and antimitotic effect.The article is suitable for Molecules. The instrument used are adequate and the statistical tests are appropriate.In my opinion, the authors described an interesting GC-MS approach to unravel new essential oils.However, there are some flaws surrounding this piece of work, which require changes in the presentation of the results.

  • Figure legends are swapped for figure 1 and 2 and figure 3 and 4.

Answer: The legend for all the figures have been exchanged and renumbered according to the suggestions of other referees. Please see the manuscript

  • I would suggest to check English spelling and removing Italian word ‘Classe’, ‘totale identificato’ in table 2.

Answer: we corrected the spelling and removed the Italian words. Please see the manuscript

  • The numbering of the table is according to their appearance in the text. Table 1 is at the end of the article. Please change.

Answer: we re-ordered the table numbers according their appearance in the text.

  • It is unclear the variation in the table 2, please provide full legend e.g. ‘H. cooperi’ (percentage%) and define percentage of identification.

Answer: the total percentage of identification has been inserted in all tables; please, see manuscript

  • Please provide more information in the figure legends.

Answer: the figure legends are already revised and are explanatory in their content. The explanation needs to be short and clear for a quick understanding of their content.

  • I would suggest to separate in new table the chemical class from the identified compounds.

Answer: we accepted your suggestion and we separated the chemical class of constituents from the identified compounds

  • Please report references for the Table 4 and 5 for previous studies.

Answer: we added the reference number for the current Table 3 and 4 (previous Table 4 and 5)

  • Table 1, which is at the end, please put as supplementary information.

Answer: table 1 is now numbered Table 5 in the revised manuscript.

In my opinion, the authors should not underestimate their results and add few more information.

Answer: Could you kindly clarify better what further information you would like to see?

The manuscript will benefit if the authors will add in the table, molecular weight and elemental composition of the identified metabolites.

Answer: manuscripts like this, on the volatile plant composition, do not usually contain “molecular weight and elemental composition of the identified metabolites”. I kindly refer you to the scientific literature of similar works (already present in the references)

  • It would be beneficial a Venn diagram where readers can appreciate the difference of the composition of the five species.

Answer: In the literature we found that this method is used in biological science to compare sets of data such as genes, proteins and so on. Venn diagrams are comprised of circles where each circle represents a whole set. Venn diagram can have unlimited circles but generally two or three circles are preferred otherwise the diagram becomes too complex. In our case we have at least four species.  If we consider them as sets, and if we use the compound as sets, we would have more than 32 sets. This is why we used Hierarchical clusteringalso known as hierarchical cluster analysis, which is an algorithm that groups similar objects into groups called clusters. The endpoint is a set of clusterswhere each cluster is distinct from each other cluster, and the objects within each cluster are broadly similar to each other.

  • Please provide in the supplement the representative total ion chromatograms for the 5 species.

Answer: We have now provided the VOC and EO chromatograms for each of the studied species with the identification of some constituents. Please see supplementary materials (Table S1 and Table S2).

  • In my opinion the manuscript will benefit from few chemical structures of the mostly changes metabolites.

Answer: As the main compounds found in the volatiles of the studied species are very well known compounds we disagree with your suggestion.  Chemical structures are unnecessary.

  • Please provide a pie chart or bar chart to illustrate the difference in compositions.

Answer:  we inserted a bar graph on the EO and SPME results (see manuscript Fig. 1 and Fig. 4)

Reviewer 2 Report

Manuscript entitled "Volatiloma analyses and in vitro antimicrobial activity of the essential oils from five South African Helichrysum species" presents the analysis of volatile organic compounds (VOCs) and essential oils (EOs) by GC-MS. Also the antimycotic activity of EOs and the antibacterial activity was studied. Both chemical and antimicrobial methods are trivial however the analysis of South African plants grown in Italy worth to be investigated and rather novel. In general manuscript is adequately written but there are some queries that need to be checked and authors should respond to them, please check below:

  1. L. 306 Living fresh plant material (1-g g) of each specie, erase g it is written two times, L. 312 material, with a weight of 3 ± 0.01 g, revise to 3.00 ± 0.01 g
  2. concerning SPME extraction, is this an in house method? if yes validation is needed, if not add reference/s. Authors say at L. 314 Exposition of the fibre took place for 15 to 30 min, you need to be specific, different times could affect absorption of compounds, what was the exact time used? Also it is not clear what was the time of desorption of compounds at the injector, this is also crucial to include. This method is a semi-quantitation method. Did you use any internal standard to check quantitation performance? Did you measure compounds in concentration or in %per cent? in Table 2 this should be stated at the title.
  3. for GC-MS which authentic samples did you use? it is not clear. Since you measure percentage why values are given as concentration? Also significant digits need to be checked e.g. 4.3±0.10 for b-pinene table 3, should be 4.30±0.10 or 4.3±0.1. all values should be expressed in the same way for all compounds.
  4. A sample chromatogram also should be presented with compounds identified, and also fragmentation for major compounds identified by MS in a table. at L. 335-336 authors say that the molecular weights of all the identified compounds were confirmed by GC/CI-MS, some results should be presented.
  5. EO yield is too low, how do you explain it? what could be the use of plants with so low EO yield?
  6. what was the solvent used for hydrodistillation according to European Pharmacopeia. add the specific reference!
  7. Table 5, how mm were measured? 
  8. Conclusions: authors say at L. 383-384: since they were rich
    in essential oils, please revise they were not rich, yields were too low
  9. General comment: why authors performed both a semi quantitative analysis like SPME and a quantitative one by hydrodistillation? Can results be compared? please comment on this

Author Response

Submission Date

22 June 2020

Date of this review

25 Jun 2020 10:14:14

Reviewer 2

Open Review

English language and style

( ) Extensive editing of English language and style required 
( ) Moderate English changes required 
(x) English language and style are fine/minor spell check required 
( ) I don't feel qualified to judge about the English language and style 

Yes

Can be improved

Must be improved

Not applicable

Does the introduction provide sufficient background and include all relevant references?

( )

( )

(x)

( )

Is the research design appropriate?

( )

( )

(x)

( )

Are the methods adequately described?

( )

( )

(x)

( )

Are the results clearly presented?

( )

( )

(x)

( )

Are the conclusions supported by the results?

( )

( )

(x)

( )

Comments and Suggestions for Authors

Manuscript entitled "Volatiloma analyses and in vitro antimicrobial activity of the essential oils from five South African Helichrysum species" presents the analysis of volatile organic compounds (VOCs) and essential oils (EOs) by GC-MS. Also the antimycotic activity of EOs and the antibacterial activity was studied. Both chemical and antimicrobial methods are trivial however the analysis of South African plants grown in Italy worth to be investigated and rather novel. In general manuscript is adequately written but there are some queries that need to be checked and authors should respond to them, please check below:

  1. L. 306 Living fresh plant material (1-g g) of each specie, erase g it is written two times,

Answer: we have deleted the latter one

  1. L. 312 material, with a weight of 3 ± 0.01 g, revise to 3.00 ± 0.01 g

Answer: We have corrected the weight according to the reviewer’s suggestion.

  1. concerning SPME extraction, is this an in house method? if yes validation is needed, if not add reference/s.

Answer: SPME extraction is a method used for a long time for this kind of experiment (evaluation of plant spontaneous emission). We have added the reference concerning the guideline for the SPME methods. Please see the manuscript (material and methods section).

  1.  Authors say at L. 314 Exposition of the fibre took place for 15 to 30 min, you need to be specific, different times could affect absorption of compounds, what was the exact time used?

Answer: The time of exposure was experimentally chosen. It depends on how intense the plant aroma was. Therefore, some samples needed only 15 min, others more and this was done in order to obtain a good chromatogram resolution to better identify the constituents.

  1. Also it is not clear what was the time of desorption of compounds at the injector, this is also crucial to include.

Answer: This time is also experimentally determined. Usually the time of the analysis (desorption time) is 30-40 min until the last compound was desorbed, evidenced in the chromatogram.  But it is reported only occasionally in the text. 

  1.  This method is a semi-quantitation method. Did you use any internal standard to check quantitation performance?

Answer: We based our study only on a semi-quantitation method (relative percentage of each constituents), as reported in many other papers and no exact quantification was done

  1. Did you measure compounds in concentration or in %per cent? in Table 2 this should be stated at the title.

Answer: the amount of the identified compounds was given in relative percentage. We added this information in all tables. Please see manuscript

  1. for GC-MS which authentic samples did you use? it is not clear. Since you measure percentage why values are given as concentration?

Answer: The identification of each compound in GC-MS was performed according to the standard procedure described in “Materials and Methods:  Chemical investigation: GC-MS analysis”.  Please see this section.

No concentration was given, only relative percentage in all the parts of the manuscript. A check of the number reported in tables 1 and 2 has been done. Commercial authentic samples were used to build the home-made mass spectra data base which we used to identify the constituents in comparison with the commercial libraries (NIST and ADAMS)

  1. Also significant digits need to be checked e.g. 4.3±0.10 for b-pinene table 3, should be 4.30±0.10 or 4.3±0.1. all values should be expressed in the same way for all compounds.

Answer: We accepted your recommendation. Please see Tables

  1. A sample chromatogram also should be presented with compounds identified, and also fragmentation for major compounds identified by MS in a table. at L. 335-336 authors say that the molecular weights of all the identified compounds were confirmed by GC/CI-MS, some results should be presented

Answer: We have now provided the VOC and EO chromatograms for each of the studied species. Usually the MS fragmentation is not reported in table (as we did for LC-MS analysis) since they are known compounds and their MS fragmentation is well known and available in the literature. The same for the molecular weight of each compound that was given by the commercial libraries and our laboratory-developed mass spectra library.

  1. EO yield is too low, how do you explain it? what could be the use of plants with so low EO yield?

Answer: It is well known in the literature that Helichrysum is a plant genus with a very good and intense smell but with a very low yield of EOs. The aim of the study was to contribute to the chemotaxonomy of these species, of which four out of five had never been studied before, and to exploit their use as ornamental plants. 

  1. what was the solvent used for hydrodistillation according to European Pharmacopeia. add the specific reference!

Answer: Hydrodistillation means distillation with water and it is the main method to obtain EO for a long time, previously inserted in the Pharmacopoeias of the different countries in the world and now included in the European Pharmacopeia (we added the reference in the proper section)

  1. Table 5, how mm were measured? 

Answer: As known in the International System of Units “mm” means millimetre used to measure the diameter of the inhibition zone in the Petri dish and we used a ruler.

  1. Conclusions: authors say at L. 383-384: since they were rich
    in essential oils, please revise they were not rich, yields were too low

Answer: These Helichrysum were rich on EO in comparison with other species of the same genus known to be less rich.

  1. General comment: why authors performed both a semi quantitative analysis like SPME and a quantitative one by hydrodistillation? Can results be compared? please comment on this

Answer: The aim of this study was to contribute to the chemotaxonomy of these South African Helichrysum spp. with the knowledge of their aroma and essential oil composition in order to exploit them for ornamental and industrial use. This is why we performed the SPME to assess their volatile emission. Afterward, although knowing that the literature reports the poverty in essential oils of this genus, we hydrodistilled the aerial parts since the essential oil of four of the five studied species have never been investigated before. These species showed not negligible yields which allowed us to assess their antimicrobial activity.

The results of the volatiles and of the EO composition are not comparable since in the first one the spontaneous emission of the plants was analysed while in the second case, we analysed an artefact product due to the heating of the plant material, and sometimes many natural products change their structure.

Reviewer 3 Report

This is an interesting manuscript showing the volatilome characterization of several essential oils from South African Helichrysum species. The experiments seem to be well performed and the description and discussion of the results are fine. However, some revision is required before the manuscript can be published in Molecules, especially regarding the journal manuscript structure.

  • The title needs to be corrected. The term “Volatiloma” is not correct in English. The correct word is “Volatilome”.

  • Please, check the affiliations of all the authors, and try to write all of them in the same format. In some of them the city is not given, or the country, etc.

  • The abstract need to be modified to follow Molecules MDPI Instructions for authors. The structure is correct, but headings (Background, Methods, Results, Conclusions) must be deleted. “The abstract should be a total of about 200 words maximum. The abstract should be a single paragraph and should follow the style of structured abstracts but without headings”

  • The provided keywords must be in italic. I will recommend also to provide some more keywords related to the methods employed, for instance, GC-MS.

Introduction:

  • Introduction its ok but it lacks information regarding the analytical methodologies typically employed for the characterization of these species. In my opinion, a paragraph commenting on that would be interesting for the readers.
  • Line 46: Correct “This species” by “These species” or “This specie”
  • Line 70: Correct “for their the volatiloma” by “for their volatilome”

Results and discussion:

  • Line 77. Table 2 is introduced here while Table 1 has not yet been introduced in the text. In my opinion, Table 2 must be labelled as Table 1.
  • Line 92: PCA and HCA need to be defined the first time they are introduced in the text.
  • Moreover, there is an error with Fig. 1 and Fig. 2 captions, they are not correct. Fig. 1 is showing the PCA loadings plot (this need to be indicated), and Fig. 2 is showing the HCA plot. These need to be corrected and the text modified accordingly. You can modify the sentence as follows: “Principal component analysis (PCA, Fig. 1) loadings plot, as well as hierarchical cluster analysis (HCA, Fig. 2) were performed for all…”.
  • Line 136. Table 3 need to be labelled as Table 2.
  • The same error is observed again with Figures 3 and 4. Figure captions are not correct. Besides, in the case of PCA, please indicate that you are depicting the loadings plot.
  • Tables 4 and 5 need to be labelled as Tables 3 and 4, respectively. Then, Table 1 (depicted as the last table in the manuscript) must be labelled as Table 5.
  • Tables 2, 3 and 5 (in the original manuscript): units need to be provided. Only in Table 4 (in the original manuscript) the authors indicated that the antifungal activity is given in %. In the other tables, the units are not provided.
  • In my opinion, it would be interesting to depict also at least 1 or 2 chromatograms of the analyzed samples showing the VOCs signals, as well as those of the standards employed for identification by comparison with retention times, as indicated by the authors. This may be provided as Supplementary Material.

Materials and methods:

  • Line 324: Electron impact is an old terminology, the correct accepted one nowadays is Electron Ionization.
  • Line 336: GC/CI-MS. Please define CI: chemical ionization

I believe that a section labelled “sample availability” is mandatory in Molecules and need to be provided after the references.

Author Response

Submission Date

22 June 2020

Date of this review

29 Jun 2020 10:16:59

Reviewer 3Inizio modulo

Open Review

English language and style

( ) Extensive editing of English language and style required 
( ) Moderate English changes required 
(x) English language and style are fine/minor spell check required 
( ) I don't feel qualified to judge about the English language and style 

Yes

Can be improved

Must be improved

Not applicable

Does the introduction provide sufficient background and include all relevant references?

( )

(x)

( )

( )

Is the research design appropriate?

(x)

( )

( )

( )

Are the methods adequately described?

(x)

( )

( )

( )

Are the results clearly presented?

( )

(x)

( )

( )

Are the conclusions supported by the results?

(x)

( )

( )

( )

Comments and Suggestions for Authors

This is an interesting manuscript showing the volatilome characterization of several essential oils from South African Helichrysum species. The experiments seem to be well performed and the description and discussion of the results are fine. However, some revision is required before the manuscript can be published in Molecules, especially regarding the journal manuscript structure.

  • The title needs to be corrected. The term “Volatiloma” is not correct in English. The correct word is “Volatilome”.

Answer:  We have corrected the term as your recommended

  • Please, check the affiliations of all the authors, and try to write all of them in the same format. In some of them the city is not given, or the country, etc.

Answer: We have written all the affiliation of all the authors in the same format and we have added the missing information

  • The abstract need to be modified to follow Molecules MDPI Instructions for authors. The structure is correct, but headings (Background, Methods, Results, Conclusions) must be deleted. “The abstract should be a total of about 200 words maximum. The abstract should be a single paragraph and should follow the style of structured abstracts but without headings”

Answer: We have adjusted the manuscript following the Author’s instructions for this journal (Molecules, MDPI). The headings were deleted and now the abstract counts 200 words

  • The provided keywords must be in italic. I will recommend also to provide some more keywords related to the methods employed, for instance, GC-MS.

Answer: All the keywords linked to the plant name are in italics according to the botanical binomial nomenclature where the names need to be written in Latinized form. The other keywords can be written in normal style.

Introduction:

  • Introduction its ok but it lacks information regarding the analytical methodologies typically employed for the characterization of these species. In my opinion, a paragraph commenting on that would be interesting for the readers.

Answer: we modified the introduction according your suggestions

  • Line 46: Correct “This species” by “These species” or “This specie”

Answer: We have corrected this word according to the reviewer's suggestion.

  • Line 70: Correct “for their the volatiloma” by “for their volatilome”

Answer: We have corrected this word according to the reviewer's suggestion. 

Results and discussion:

  • Line 77. Table 2 is introduced here while Table 1 has not yet been introduced in the text. In my opinion, Table 2 must be labelled as Table 1.

Answer: we have modified the Table numbers

  • Line 92: PCA and HCA need to be defined the first time they are introduced in the text.

Answer: The full mane of abbreviations was added in this line. Please see the manuscript

  • Moreover, there is an error with Fig. 1 and Fig. 2 captions, they are not correct. Fig. 1 is showing the PCA loadings plot (this need to be indicated), and Fig. 2 is showing the HCA plot. These need to be corrected and the text modified accordingly. You can modify the sentence as follows: “Principal component analysis (PCA, Fig. 1) loadings plot, as well as hierarchical cluster analysis (HCA, Fig. 2) were performed for all…”.

Answer: we have edited the figure captions. Please see the manuscript.

  • Line 136. Table 3 need to be labelled as Table 2.

Answer:  We have re-labelled the EO and VOC tables in the same manner and we have also added more info in the footnote. Please see the manuscript.

  • The same error is observed again with Figures 3 and 4. Figure captions are not correct. Besides, in the case of PCA, please indicate that you are depicting the loadings plot.

Answer: We have also accepted this revision.

  • Tables 4 and 5 need to be labelled as Tables 3 and 4, respectively. Then, Table 1 (depicted as the last table in the manuscript) must be labelled as Table 5.

Answer: We have corrected the table sequence. Please see the manuscript.

  • Tables 2, 3 and 5 (in the original manuscript): units need to be provided. Only in Table 4 (in the original manuscript) the authors indicated that the antifungal activity is given in %. In the other tables, the units are not provided.

Answer: Tables were renumbered according their appearance in the manuscripts.  We have added the measurement unit (%) in tables 1a-b, and 2a-b.  In table 4 the units were already present: Disk diffusion (mm) and MIC (%).

  • In my opinion, it would be interesting to depict also at least 1 or 2 chromatograms of the analyzed samples showing the VOCs signals, as well as those of the standards employed for identification by comparison with retention times, as indicated by the authors. This may be provided as Supplementary Material.

Answer: We have inserted one chromatogram for each VOC and EO analysis of the four plant samples as Supplementary materials (Table S1-S2). The identification method is already reported in the ““Materials and Methods:  Chemical investigation: GC-MS analysis”. 

Materials and methods:

  • Line 324: Electron impact is an old terminology, the correct accepted one nowadays is Electron Ionization.

Answer: we have corrected the word as suggested by the reviewer.

  • Line 336: GC/CI-MS. Please define CI: chemical ionization

Answer: We have added the definition of the abbreviation  

I believe that a section labelled “sample availability” is mandatory in Molecules and need to be provided after the references.

Answer: we didn’t find any paragraph linked to the “sample availability” in the Molecules instructions for authors. However, the vouchers of the studied plants are reported in table 5 (Please, see manuscript). Fresh plant materials are also available and conserved at CREA (Sanremo, Italy)

Round 2

Reviewer 2 Report

Authors replied adequately to my comments and queries concerning their work. Now paper is more clear to readers. At least one indicative VOC and EO chromatogram should be present within manuscript and not just as supplement material. Also MS fragmentation is important since not all laboratories have access to Nist or wiley libraries (however not need to include now). Still it is not clear which were the standard compounds used to prepare libraries, these should be stated and from which company they were purchased.

Minor spelling and grammar mistakes.

Author Response

Reviewer 2

Open Review

English language and style

( ) Extensive editing of English language and style required 
( ) Moderate English changes required 
(x) English language and style are fine/minor spell check required 
( ) I don't feel qualified to judge about the English language and style 

Yes

Can be improved

Must be improved

Not applicable

Does the introduction provide sufficient background and include all relevant references?

(x)

( )

( )

( )

Is the research design appropriate?

(x)

( )

( )

( )

Are the methods adequately described?

( )

(x)

( )

( )

Are the results clearly presented?

(x)

( )

( )

( )

Are the conclusions supported by the results?

(x)

( )

( )

( )

Comments and Suggestions for Authors

Authors replied adequately to my comments and queries concerning their work. Now paper is more clear to readers. At least one indicative VOC and EO chromatogram should be present within manuscript and not just as supplement material.

Answer: In our opinion it is sufficient to insert all the chromatograms in the supplementary material instead of one of them in the text as an example.

Also MS fragmentation is important since not all laboratories have access to Nist or wiley libraries (however not need to include now).

Answer: we understand your criticism, but to include now the MS fragmentation of all the compounds is very time consuming and not necessary since the majority of the people, who are working in this field, know them very well.

Still it is not clear which were the standard compounds used to prepare libraries, these should be stated and from which company they were purchased.

Answer: we accepted your recommendation and we inserted more information in the table with footnote concerning the authentic compounds used for the identification of the constituents. Please see Table 1.a and 2.a 

Minor spelling and grammar mistakes.

Answer: The manuscript was revised by an English mother language

Please, note that we also changed some photos with other clearer and more done by one of us (Claudio Cervelli)

Reviewer 3 Report

The quality of the manuscript was improved by following reviewers' suggestions, and it is acceptable for publication.

Author Response

Reviewer 3

Open Review

English language and style

( ) Extensive editing of English language and style required 
( ) Moderate English changes required 
( ) English language and style are fine/minor spell check required 
(x) I don't feel qualified to judge about the English language and style 

Yes

Can be improved

Must be improved

Not applicable

Does the introduction provide sufficient background and include all relevant references?

(x)

( )

( )

( )

Is the research design appropriate?

(x)

( )

( )

( )

Are the methods adequately described?

(x)

( )

( )

( )

Are the results clearly presented?

(x)

( )

( )

( )

Are the conclusions supported by the results?

(x)

( )

( )

( )

Comments and Suggestions for Authors

The quality of the manuscript was improved by following reviewers' suggestions, and it is acceptable for publication.

Answer: Thank you
