# Supplementary material for: Volatilome Analyses and In Vitro Antimicrobial Activity of the Essential Oils from Five South African Helichrysum Species
Source: Molecules. 2020 Jul 13;25(14):3196. doi: 10.3390/molecules25143196 (PMC7397169; doi:10.3390/molecules25143196)
Supplement: Supplementary file 1 [file molecules-25-03196-s001.pdf]

Supplementary Materials

Volatilome Analyses and In Vitro Antimicrobial Activity of the Essential Oils from Five South African *Helichrysum* Species

Table S1. VOC chromatograms of the studied *Helichrysum* spp. with the main compounds.

<table>
<tr><td>
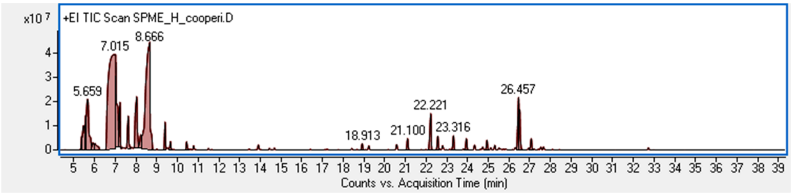

</td><td>

A: H. cooperi

5.659: α-pinene
7.015: sabinene
8.666: 1,8-cineole
26.457: γ-muurolene
</td></tr>
<tr><td>
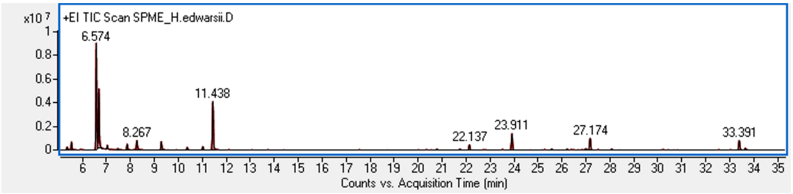

</td><td>

B: H. edwarsii

6.571: sabinene
11.438: trans-thujone
23.915: β-caryophyllene
</td></tr>
<tr><td>
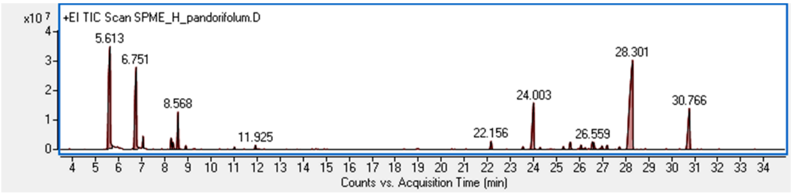

</td><td>

C: H. pandorifolium

5.613: α-pinene
6.751: beta-pinene
24.003: β-caryophyllene
28.301: δ-cadinene
30.766: viridiflorol
</td></tr>
<tr><td>
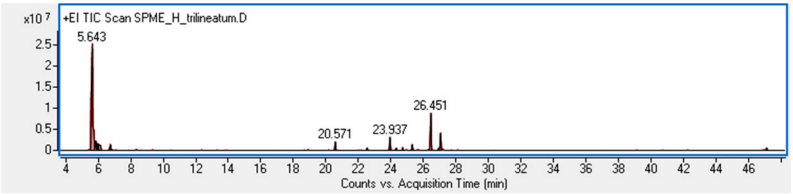

</td><td>

D: H. trilineatum

5.643: α-Pinene
20.571: δ-eIemene
23.937: β-caryophyllene
26.451: germacrene D
</td></tr>
</table>

Table S2: EO chromatograms of the studied *Helichrysum* spp. with the main compounds.

<table>
<tr><td>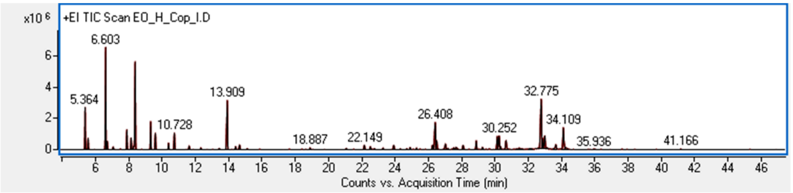
</td>
<td>A: H. cooperi

5.364: α-thujene
6.603: sabinene
13.909: terpinen-4-ol
26.408: germacrene D
32.775: himachalol
34.109: epi-α-bisabolol</td></tr>
<tr><td>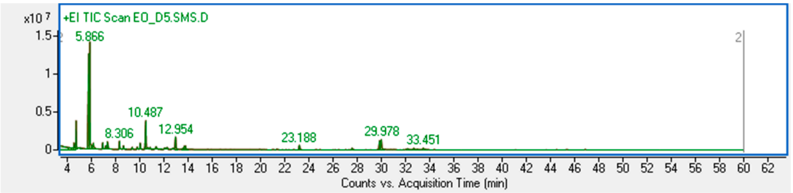
</td>
<td>B: H. edwarsii

5.866: sabinene
10.487: trans-thujone</td></tr>
<tr><td>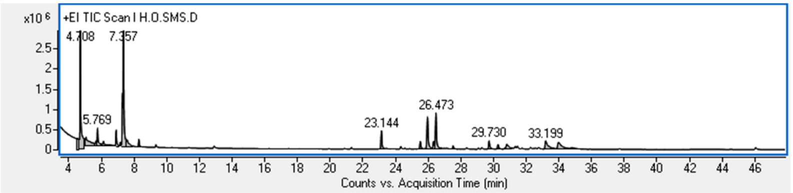
</td>
<td>C: H.odoratissimum

4.708: α-pinene
7.357: 1,8-cineol
23.144: β-caryophyllene
26.473: epi-cubebol</td></tr>
<tr><td>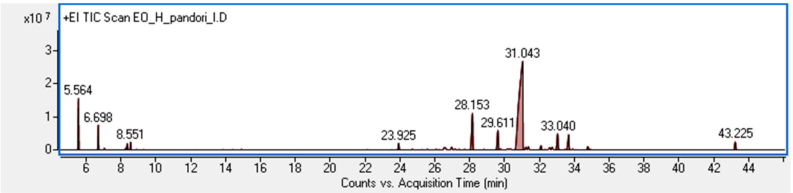
</td>
<td>D: H. pandorifolium

5.564: alpha pinene
28.153: δ-cadinene
31.043: Viridiflorol
33.040: pogostol</td></tr>
<tr><td>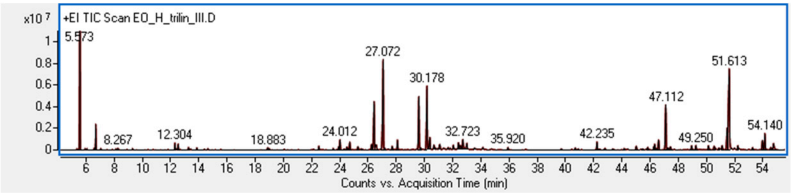
</td>
<td>E: H. trilineatum

5.573: α-pinene
27.072: bicyclogermacrene
30.178: spathulenol
47.112: Abieta-7,13-diene
51.613: sandaracopimarinol</td></tr>
</table>
